# Supplementary material for: Construction and Mechanism Analysis of a Self-Assembled Conductive Network in DGEBA/PEI/HRGO Nanocomposites by Controlling Filler Selective Localization
Source: Nanomaterials (Basel). 2021 Jan 16;11(1):228. doi: 10.3390/nano11010228 (PMC7830563; doi:10.3390/nano11010228)
Supplement: Supplementary file 1 [file nanomaterials-11-00228-s001.pdf]

Supplementary Materials

Construction and Mechanism Analysis of a Self-Assembled Conductive Network in DGEBA/PEI/HRGO Nanocomposites by Controlling Filler Selective Localization

Yiming Meng [1,2], Sushant Sharma [1], Wenjun Gan [2], Seung Hyun Hur [1], Won Mook Choi [1] and Jin Suk Chung [1,*]

Table S1. Formulation of samples.

Sample	DGEBA (phr)[1]	PEI (phr)[1]	HRGO (phr)[1]	C–A solution
DGEBA	100	0	0	80
DP0H	100	0	(100 + 0) × 0.5%	80
DP5H	100	5	(100 + 5) × 0.5%	80
DP10H	100	10	(100 + 10) × 0.5%	80
DP15H	100	15	(100 + 15) × 0.5%	80
DP20H	100	20	(100 + 20) × 0.5%	80
DP25H	100	25	(100 + 25) × 0.5%	80
DP30H	100	30	(100 + 30) × 0.5%	80

[1]phr: parts per hundred in the DGEBA.

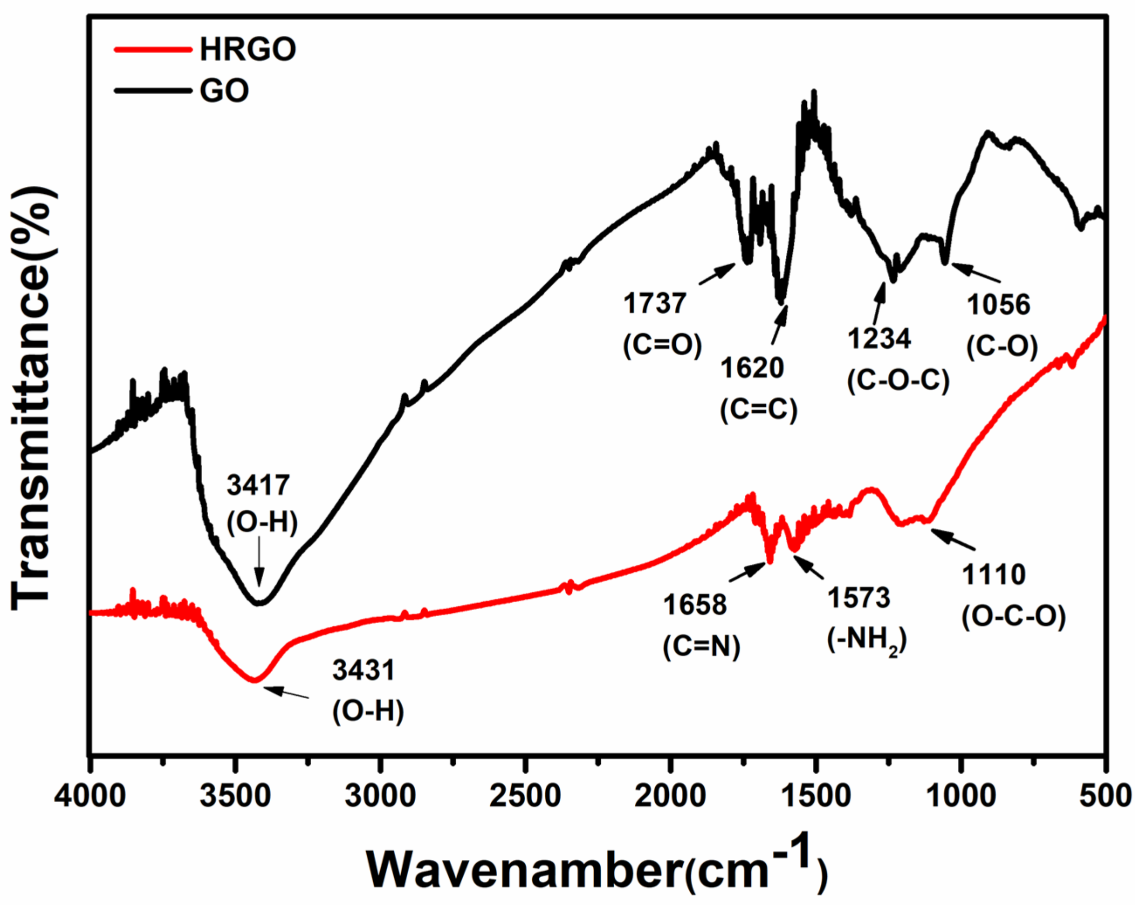

Figure S1. The FTIR spectra for HRGO and GO.

The reduction of GO was determined using FTIR technique. The FTIR spectra of HRGO and GO are represented

in Figure S1. The broad peak from ~ 3000 to ~3600 cm^{-1} was associated to the hydroxyl group (from -COOH and H_2O), which reduces in case of HRGO [S3]. The peaks at ~1737, ~1620,~1234 and 1056 cm^{-1} are associated with the stretching modes of ketone C=O, sp^2 hybridized carbon in plane C=C, epoxide C-O-C and alkoxy C-O [S4, S5]. However, in the case of HRGO, a significant decrement in peak intensities was observed and few peaks at ~1658 cm^{-1} and 1573 cm^{-1} were observed. The peaks of ~ 1658 cm^{-1} and 1573 cm^{-1} are formed due to hydrazine reduction and basically associated with C=N and $-NH_2$ stretching[S6,S7].

Table S2. Surface tensions and components (mJ/m^2) suggested by van Oss et al.[S1,S2]

Liquid	γ_L	$\gamma_L^{LW}(\gamma_L^d)$	$\gamma_L^{AB}(\gamma_L^P)$	γ_L^+	γ_L^-	Polarity[γ_L^P/γ_L^d]
Deionized water (DI)	72.8	21.8	51	25.5	25.5	2.3
Glycerol (GL)	64.0	34.0	30	3.9	57.4	0.9
Formamide (FA)	58.0	39.0	0	2.28	39.6	0

Table S3. The static contact angles for the test liquids DGEBA, PEI, and HEGO.

Liquid	Contact angle(θ)		
	DGEBA	PEI	HRGO
Deionized water (DI)	88.4	84.2	80.5
Glycerol (GL)	84.3	75.0	55.0
Formamide (FA)	54.1	58.8	27.1

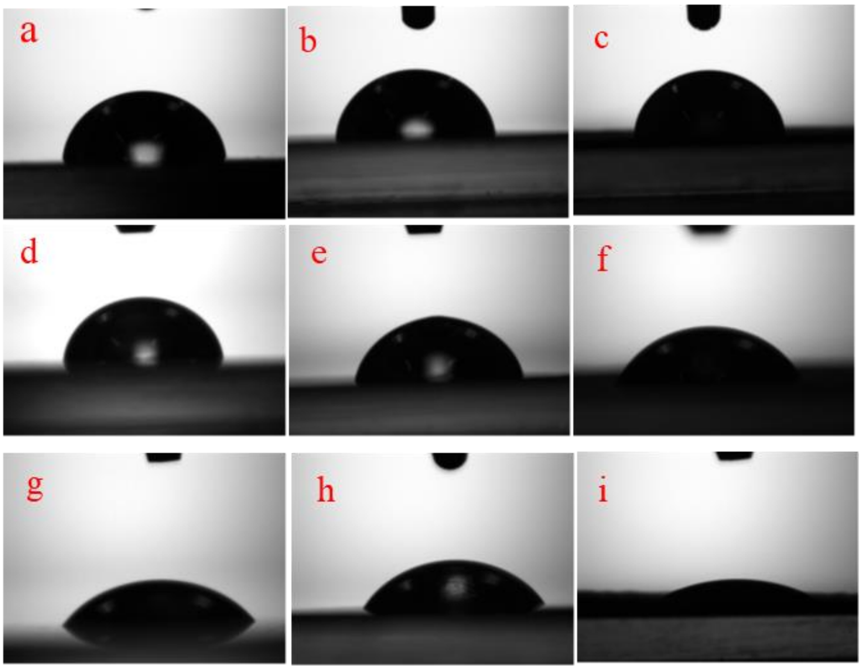

Figure S2. The contact angle images of samples given in Table S3. (a) DI on DGEBA, (b) DI on PEI, (c) DI on HRGO, (d) GL on DGEBA, (e) GL on PEI, (f) GL on HRGO, (g) FA on DGEBA, (h) FA on PEI, (i) FA on HRGO.

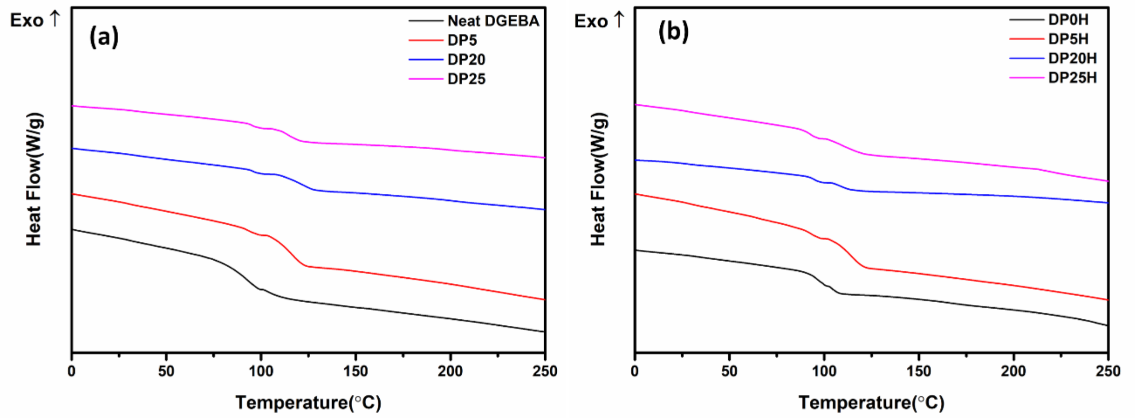

Figure S3. The differential scanning calorimeter (DSC) curves of (**a**) DGEBA/PEI polyblends and (**b**) DGEBA/PEI/HRGO polyblends.

References

[S1] Van Oss, C.; Good, R.; Chaudhury, M. The role of van der Waals forces and hydrogen bonds in "hydrophobic interactions" between biopolymers and low energy surfaces. *Journal of colloid and Interface Science* **1986**, *111*, 378-390.

[S2] Li, Z.; Giese, R.; Van Oss, C.; Yvon, J.; Cases, J. The surface thermodynamic properties of talc treated with octadecylamine. *Journal of colloid and interface science* **1993**, *156*, 279-284.

[S3] Acik, M.; Lee, G.; Mattevi, C.; Pirkle, A.; Wallace, R.M.; Chhowalla, M.; Cho, K.; Chabal, Y. The role of oxygen during thermal reduction of graphene oxide studied by infrared absorption spectroscopy. *The Journal of Physical Chemistry C* **2011**, *115*, 19761-19781.

[S4] Das, A.K.; Srivastav, M.; Layek, R.K.; Uddin, M.E.; Jung, D.; Kim, N.H.; Lee, J.H. Iodide-mediated room temperature reduction of graphene oxide: a rapid chemical route for the synthesis of a bifunctional electrocatalyst. *Journal of Materials Chemistry A* **2014**, *2*, 1332-1340.

[S5] Fan, B.; Guo, H.; Shi, J.; Shi, C.; Jia, Y.; Wang, H.; Chen, D.; Yang, Y.; Lu, H.; Xu, H. Facile one-pot preparation of silver/reduced graphene oxide nanocomposite for cancer photodynamic and photothermal therapy. *Journal of Nanoscience and Nanotechnology* **2016**, *16*, 7049-7054.

[S6] Gong, Y.; Li, D.; Fu, Q.; Pan, C. Influence of graphene microstructures on electrochemical performance for supercapacitors. *Progress in Natural Science: Materials International* **2015**, *25*, 379-385.

[S7] Zhong, B.; Shen, L.; Zhang, X.; Li, C.; Bao, N. Reduced graphene oxide/silica nanocomposite-reinforced anticorrosive fluorocarbon coating. *Journal of Applied Polymer Science* **2021**, *138*, 49689.
